# Physiological Regulation of Growth, Hematology and Blood Gases in Chicken Embryos in Response to Low and High Incubation Humidity

**DOI:** 10.3389/fphys.2022.880737

**Published:** 2022-05-24

**Authors:** Sylvia Branum, Hiroshi Tazawa, Warren Burggren

**Affiliations:** Developmental Integrative Biology, Department of Biological Sciences, University of North Texas, Denton, TX, United States

**Keywords:** chicken embryo, incubation, relative humidity, embryonic development, acid-base balance, hematology, hydration, dehydration

## Abstract

Variations from a relative humidity (RH) of ∼50–60% can unfavorably alter chicken embryo development, but little is known of whether the embryo can mitigate these effects through physiological regulation. We examined effects of Low RH (25–35%), and High RH (85–93%) compared to Control RH (50–60%) on hatchability, embryonic growth, hematology and blood gases and pH. Mean hatchability was not affected by RH. Yet, Low RH decreased wet body mass of advanced embryos (days 17–19; d17-19), with lowered body water content compared with embryos of Control and High RH. However, dry body mass of developing (d11-19) embryos was not different between the three RH groups. Mean blood osmolality across development was higher in Low RH embryos and lower in High RH embryos compared with Control embryos. Mean blood lactate was higher in both Low and High RH embryos compared to Control embryos. Unexpectedly, hematological respiratory variables (Hct, [RBC], MCV, [Hb]) and blood gas variables (Po_2_, Pco_2_, pH, [HCO_3_^−^]) across development were not affected by RH. Mean wet body mass at hatch (d20-22) was larger in High RH embryos compared with Low RH embryos, but mean wet and dry body mass upon euthanasia on d22 was unaffected. The ability of the three populations to physiologically regulate blood respiratory variables and blood acid-base balance was then examined by observing their responses to intrinsic hypoxemia and hypercapnia created by controlled partial egg submersion in water. Hct and [RBC] responses were less disturbed by submersion in High RH embryos compared with both Control and Low RH embryos, which showed major disturbance. Acid-base regulatory responses did not differ between RH groups. We conclude that, while different incubation RHs cause large differences in tissue water content and body mass, most hematological and acid-base regulatory capabilities are regulated near Control values.

## 1 Introduction

Chicken eggs are typically incubated at ∼37–38°C of temperature and a ∼50–60% relative humidity (RH). Deviation of incubation temperature and/or humidity from these standard ranges produces abnormal embryonic development and hatchability (Noiva et al., 2014). While major deviations in incubation temperature are invariably lethal, it is less likely that even large deviations of RH created during artificial incubation causes total mortality, because embryos in the egg are protected by the finite water vapor conductance created by the eggshell. In typical desktop (non-commercial) egg incubators, RH falls no lower than ∼20–30% (Low RH) or higher than ∼85–93% (High RH). Even these relatively dry or wet conditions are not always lethal, but mortality is much increased in comparison with incubation at RH of ∼50–60% (Control) due ultimately to the degradation of embryonic physiology. A classic study showed that estimated mortality in chicken embryos increasing to 1/3—½ of all eggs incubated at a RH of ∼30–20% and to ½-3/4 incubated at a highly elevated RH of ∼85–93% (Ar and Rahn, 1980). These values compare to estimated minimal mortality of ∼1/5 in Control eggs incubated at 50–60% RH. Even when chicken embryos survived these extreme incubation RHs, some physiological functions are likely to be degraded even as others are strengthened to cope with altered RH. In an example of enhanced physiological function to meet this environmental challenge, presumably advantageous renal morphological and physiological remodeling occurs in chicken embryos surviving incubation in low humidity (Bolin et al., 2017).

In the current study, we examined potential effects of both elevated and reduced RH on embryonic growth, hematology, respiratory blood gases and acid-base variables. We created an additional physiological challenge by transient, partial water submergence in d17 eggs to judge the efficiency of physiological regulation in altered humidity conditions. We first hypothesized that, predictably, d17-21 embryos incubated in High RH will be hydrated and Low RH will be dehydrated, with lower hatchability in both groups compared with Control embryos. Additionally, we hypothesized that embryos incubated in High and Low RH will show altered physiological regulatory capabilities that could enhance survival.

## 2 Materials and Methods

### 2.1 Egg Incubation

All experiments with chicken embryos were performed at the University of North Texas in accordance with the protocol approved by the UNT Institutional Animal Care and Use Committee. Fertile eggs of the chicken (*Gallus gallus domesticus*, layer strain) were obtained weekly from a local hatchery. Upon arrival at the laboratory, eggs were weighed to 0.01 g with an electronic balance. Very small (<50 g) or very large (>70 g) eggs were excluded from the study. Eggs were numbered in order of egg mass and divided equally into the three groups according to egg mass. The eggs in individual groups were placed at 12:00 in three Hova-Bator desk-top incubators (GQF Manufacturing, Savannah, Georgia) set to a temperature of 38 ± 0.5°C. Control eggs were incubated at ∼50–60% RH (Control RH), while other two groups were incubated at ∼20–35% RH (Low RH) and ∼85–93% RH (High RH), respectively. The Low RH conditions was created by providing no water to the incubator, while the High RH was created by filling all available pools in the incubator with water. Relative humidity was monitored by Baro-Thermo-Hydrometers (Oregon Scientific, Tualatin, OR, United States).

This study was completed in a series of six incubation experiments between February 15^th^ and November 20^th^ in the same calendar year. Table 1 outlines these experiments, providing n numbers and additional information on hatchability.

**TABLE 1:** Hatchability of eggs incubated in Low, Control and High relative humidity determined from six separate experiments.

		Hatching variable	Egg set date						
			Feb. 15^th^	Apr. 30^th^	Sep. 19^th^	Oct. 9^th^	Oct. 29^th^	Nov. 20^th^	Mean ± se
Relative Humidity (%)	CONTROL (50–60%)	# of Eggs Incubated	28	28	28	28	28	28	−
		# of Fertilized Eggs	24	23	23	23	21	18	22 ± 1
		# of Hatchlings	18	17	20	16	13	9	16 ± 2
		Hatchability of Set Eggs (%)	64%	61%	71%	57%	46%	32%	55 ± 6%
		Hatchability of Fertilized Eggs (%)	75%	74%	87%	70%	62%	50%	70 ± 5%
	LOW (25–35%)	# of Eggs Incubated	28	28	28	28	28	28	−
		# of Fertilized Eggs	19	20	20	15	21	23	20 ± 1
		# of Hatchlings	10	14	13	7	15	12	12 ± 1
		Hatchability of Set Eggs (%)	36%	50%	46%	25%	54%	43%	42 ± 4%
		Hatchability of Fertilized Eggs (%)	53%	70%	65%	4%7	71%	52%	60 ± 4%
	HIGH (85–93%)	# of Eggs Incubated	28	28	28	28	28	28	−
		# of Fertilized Eggs	24	22	22	24	21	19	22 ± 1
		# of Hatchlings	16	18	13	15	15	15	15 ± 1
		Hatchability of Set Eggs (%)	57	64	46	54	54	54	55 ± 2%
		Hatchability of Fertilized Eggs (%)	67	82	59	63	71	79	70 ± 4%

Water loss from eggs was determined as the difference of egg mass from d0 to a given day.

### 2.2 Protocols

Examination of incubation RH effects focused on three categories of variables: hatching success, changes of physiological variables during the developmental process, and physiological regulatory capabilities in the face of environmental challenges (intrinsic progressive hypercapnia and hypoxia). On the day of experiment, individual egg mass was measured in the morning prior to blood sampling. Individual embryos were then euthanized after blood sampling and measured for wet and dry body mass.

#### 2.2.1 Hatchability

Fertile eggs with growing embryos were confirmed by candling on d4 of incubation and continuously incubated in their assigned RH level. Hatchability was determined as the percentage of successful hatchlings from the initial fertile eggs.

#### 2.2.2 Hatchling Wet and Dry Body Mass

On d19 of incubation, the viable eggs in the three RH groups were measured for mass to nearest 0.01 g. Water loss from the beginning of the incubation period was determined. The eggs in the three RH groups were transferred sequentially to hatchers consisting of desk-top incubators provided with 25 partitions equally divided by cardboard partitions and maintained at ∼37°C and ∼60–65% RH. These standardized hatching conditions were created to ensure that all embryos hatched under the same conditions, thus removing effects of different environmental humidity conditions during the hatching process. This assumed that any permanent changes related to development in high or low RH that had appeared during the entire process of development would not be quickly reversed during a final day leading up to hatching.

Hatchers were monitored every h from 0:700 to 21:00 every d until d22 to obtain newly hatched chicks and determine their wet body mass to the nearest 0.01 g. On d22, hatchlings were transferred to a closed container and euthanized with isoflurane, immediately followed by determination of wet body mass to the nearest 0.01 g. The carcasses were then placed on tared plastic trays and placed in a desiccating oven at 65°C. The dry body mass was determined when the mass of body and tray changed by < 0.01 g during three consecutive days of dry mass measurement. Typically, this occurred after ∼17–19 days of drying in the oven.

#### 2.2.3 Daily Water Loss

During the period from incubation d11 to d19, water loss from individual embryonated eggs on the target day was determined from the egg mass difference between d0 and the morning of the day prior to blood sampling.

#### 2.2.4 Blood Sampling

The allantoic vein was located by candling and the location marked on the eggshell 1 day before the target developmental day. On the target developmental day, a ∼0.8 cm square of eggshell was removed at the marked region. The now-exposed underlying allantoic vein was gently lifted by forceps through the hole in the eggshell. Arterialized blood from the allantoic vein was drawn into a 25-gauge needle attached to a 1 ml sampling syringe which had been flushed in advance with heparinized saline. 200 µL of blood was withdrawn on d11, and 250 µL of blood withdrawn on each of all subsequent days. Since the allantoic vein of d19 embryos could not be candled accurately, arterialized blood was not always collected on d19.

Once blood had been collected, the sampled eggs were placed for at least 30 min into an anoxic bag vented with N_2_ to euthanize the embryos. Embryos minus allantoic membranes were then removed from the eggs, blotted with paper to remove surface fluids and wet body mass (WBM) measured to the nearest 0.01 g. Dry body mass was then determined as described above for embryos. Body water content (% of embryo body) was calculated as: Water content = 100 x (WBM—DBM)/WBM.

#### 2.2.5 Hematology and Blood Gases

Immediately after collection, blood was gently transferred to a 0.15 ml plastic vial. From this vial ∼120 µL of blood was withdrawn and injected into a blood gas analyzer (ABL5, Radiometer Medical A/S, Copenhagen, Denmark) to determine blood Po_2_ (mmHg), Pco_2_ (mmHg), pH and [HCO_3_^−^] (mmol L^−1^). The blood remaining in the sampling syringe was transferred to the vial, thoroughly mixed and then injected in a blood cell counter (Coulter analyzer, A^c^·10T, Beckman, United States) to measure red blood cell concentration ([RBC], 10^6^ μL^−1^) and hemoglobin concentration ([Hb], g%). A further ∼10 µL of blood was used to measure blood osmolality (Osm, mmol kg^−1^) using a vapor pressure osmometer (Vapro 5520, Wescor, United States). With one drop of blood lactate ion concentration ([La^−^], mmol L^−1^) was determined with a lactate meter (Nova Lactate Plus Meter, Nova Biomedical, MA, United States). Finally, ∼60 µL of blood was transferred into each of two hematocrit tubes. The tubes were sealed and centrifuged for 5 min at 10,000 rpm to determine hematocrit (Hct, ±0.1%) (Readacrit Centrifuge, Becton Dickinson, United States). The mean value of duplicate determinations was Hct for this embryo. From the measured values of Hct, [RBC] and [Hb], the following mean corpuscular variables were calculated; mean corpuscular volume (MCV = 10 x Hct/[RBC] in µm^3^), mean corpuscular hemoglobin (MCH = 10 x [Hb]/[RBC] in pg) and mean corpuscular hemoglobin concentration ([MCHb] = 100 x [Hb]/Hct in g%).

Blood acid-base status was represented on a Davenport diagram, constructed by plotting Pco_2_ isopleths calculated from the Henderson-Hasselbalch equation using a CO_2_ solubility factor of 0.0308 mmol L^−1^ mmHg^−1^ and a serum carbonic acid pK’ according to pH (Severinghaus et al., 1956a, b). A mean slope of -16 mmol L^−1^ pH^−1^ was used for the buffer line of chicken embryo blood (Burggren et al., 2012).

#### 2.2.6 Experimental Challenge to Assess Physiological Regulation of Hematological and Acid-Base Disturbances

Embryos under the various RH incubation regimes were assessed for altered regulation of hematological variables, blood respiratory gases and acid-base balance when experimentally exposed to progressive intrinsic hypoxia and hypercapnia. These conditions were created by partial egg submersion in water, previously established as a method for testing embryonic physiological regulation (Andrewartha et al., 2014; Branum et al., 2016). On d16 of incubation, the viable eggs in the three RH groups had a red line drawn on the egg’s mid-line. Details of the submersion process are described in Andrewartha et al. (2014) and Branum et al. (2016). Each RH group was divided into four subgroups; 0 h (Control without water submersion), 2, 6 and 24 h of submersion. Submersion of the 24 h group began on d16 so that it would end on d17, as for the other groups. All eggs were submersed with air cell down.

After the designated time of water submersion (2, 6 and 24 h), each egg was removed from the water and the submerged half of the egg was immediately covered with Parafilm to preserve blood gas values during blood collection by continuing to eliminate gas exchange across the egg shell between environment and air cell. An area ∼1 cm across was created in Parafilm over the site of the allantoic vein and a small hole ∼0.8 cm across opened in the eggshell. Arterialized blood was immediately collected and analyzed for Pco_2_, pH and [HCO_3_^−^] with the blood gas analyzer and subsequently analyzed for all of the hematological variables described above.

### 2.3 Statistical Analysis

Data were tested for normality and equal variance. Differences between means of multiple groups were tested by one-way ANOVA with post-hoc multiple comparison analysis to determine differences between individual group means. Multiple group means consisting of several subsets individually were examined by two-way ANOVA for the significance of difference between group means and between individual subsets. Significance was assumed at *p* < 0.05. All data were presented as means ±1 S.E.M.

## 3 Results

### 3.1 Hatchability and Hatchling Gross Morphology

Overall hatchability was 70 ± 5% for fertilized Controls (*n* = 132), 60 ± 4% for Low RH (*n* = 118) and 70 ± 4% High RH (*n* = 132) (Table 1). There were no significant differences in % hatchability between the three RH groups (*p* = 0.203). There was also no significant relationship between incubation time and hatching time between the three experimental groups.

While hatchlings were not subjected to necropsy, we observed no gross morphological developmental disturbances, such as wing, beak or limb abnormalities, edema or abnormal yolk sac resorption.

### 3.2 Daily Egg Water Loss

**FIGURE 1:**
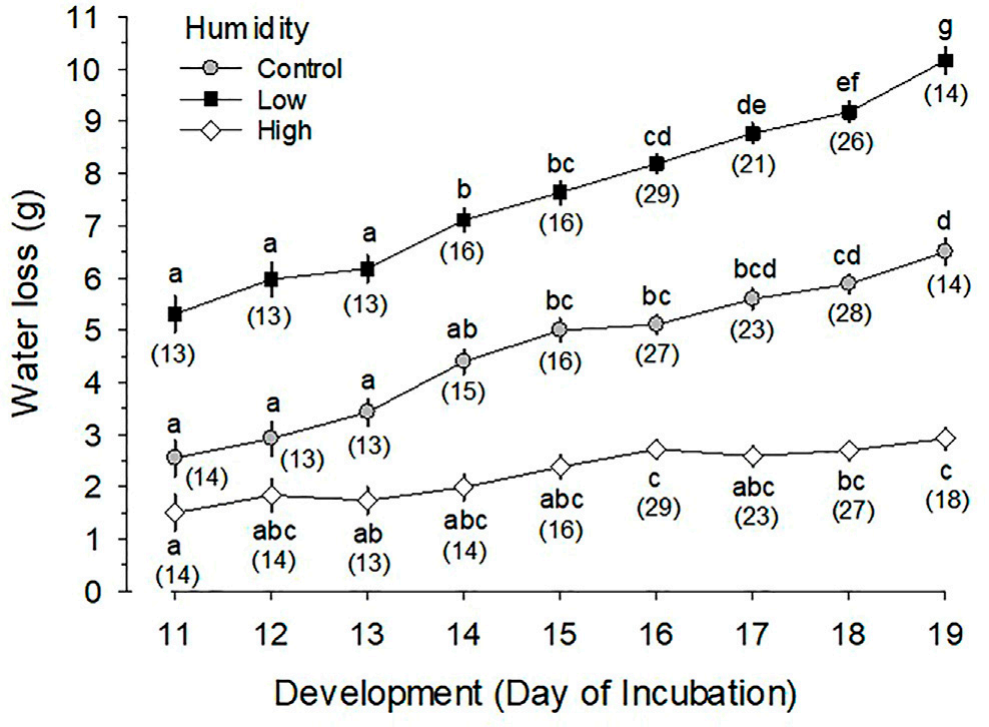
Daily water loss (g) from eggs incubated at Control (∼50–60%), Low (∼25–30%) and High (∼80–93%) relative humidity during the last half of egg incubation. Different letters for means in each of the three relative humidity groups indicate significant differences, while number of measurements (n) is shown in parentheses. Mean values at any given day of incubation (i.e., vertical comparison) for each of the three relative humidities are significantly different from each other (*p* < 0.001). Daily water loss (WL in g) is expressed by the following linear regression equations; Low RH, WL = 0.59·Day-1.19 (*p* < 0.001, *t* = 14.17 for coefficient); Control RH, WL = 0.46·Day-2.22 (*p* < 0.001, *t* = 13.69); and High RH, WL = 0.17·Day-0.32 (*p* < 0.001, *t* = 9.03).

Egg water loss during d11 to 19 of incubation significantly increased with development (*p* < 0.001) and was significantly different between the three RH groups (*p* < 0.001) (Figure 1). Average water loss during the designated period of incubation was 7.61 ± 0.08 g (13.5 ± 0.1% of egg mass) for Low RH, 4.66 ± 0.08 g (8.2 ± 0.1%) for Control RH, and 2.26 ± 0.08 g (4.0 ± 0.1%) for High RH. On d19 of incubation, the average water loss was 10.17 ± 0.24 g (17.6 ± 0.4%), 6.50 ± 0.24 g (11.2 ± 0.4%) and 2.92 ± 0.21 g (5.0 ± 0.4%) for Low, Control and High RH, respectively.

### 3.3 Wet and Dry Body Mass of Hatchlings

#### 3.3.1 Wet Body Mass

**FIGURE 2:**
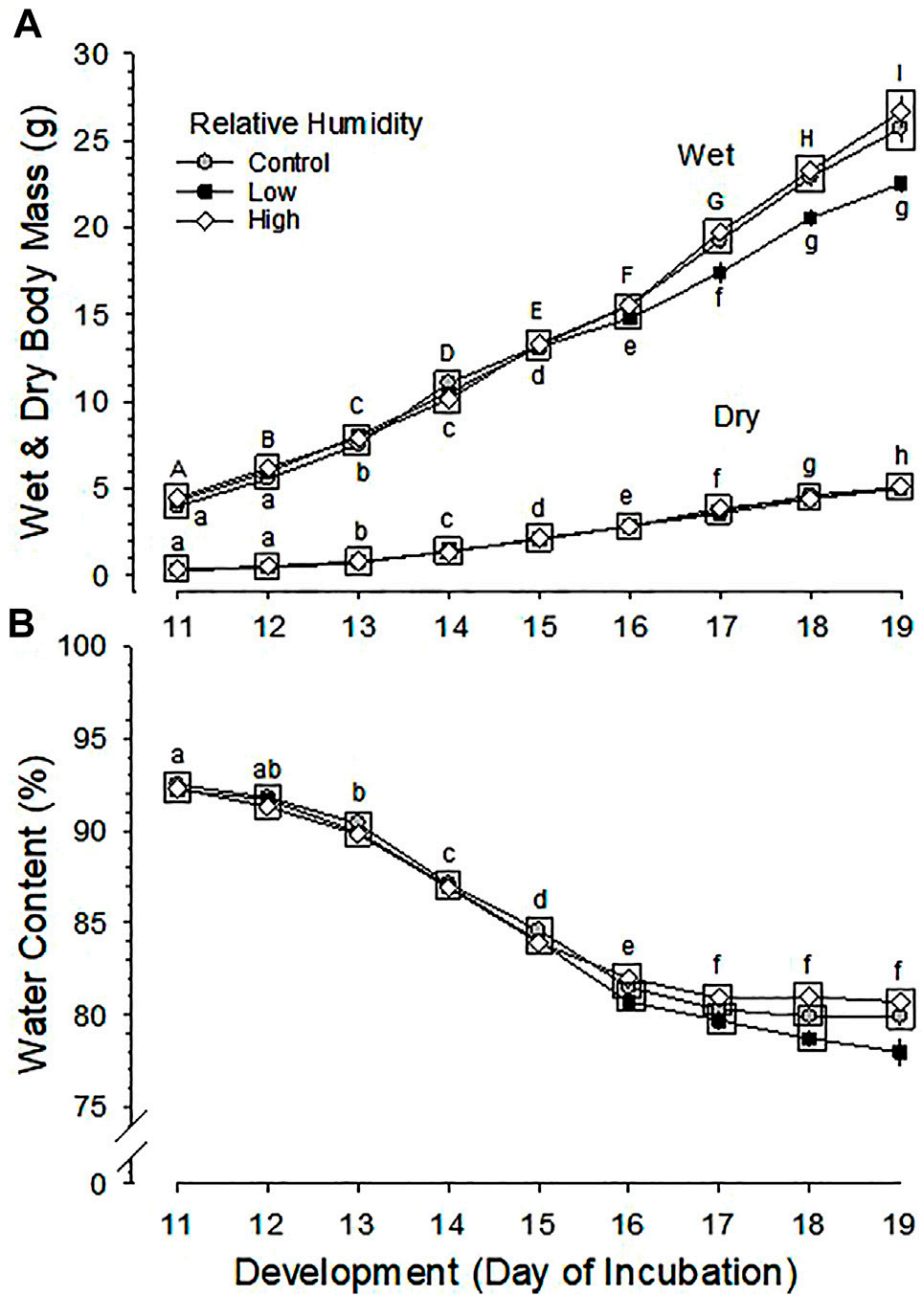
Developmental changes in **(A)** wet and dry body mass and **(B)** body water content of embryos incubated at Control, Low and High relative humidity. Means ± standard error are presented. In most cases error bars are smaller than the symbol size. Means on any given day of incubation that are not significantly different from each other are enclosed by a box. **(A)** Wet and Dry Body Mass: Effect of RH and development on wet body mass and their interaction are all significant (*p* < 0.001 for all). Different uppercase letters indicate significant difference between development of embryos incubated at Control and High RH and lowercase letters indicate significant difference between development of embryos incubated at Low RH. Effect of development is significant (*p* < 0.001), but there is no significant effect of RH on dry body mass of the three groups (*p* = 0.695), and with no significant interaction between RH and development (*p* = 0.877). **(B)** Body Water Content: Effect of RH, development and their interaction on water content are all significant (*p* < 0.001 for all). Lowercase letters indicate significant differences between developments of embryos incubated at Control RH. *n* values are as in Figure 1.

Developmental changes in wet body mass were significantly different between the three RH groups (*p* < 0.001 for effect of humidity) and were significant (*p* < 0.001 for development) with significantly different rate between groups (*p* < 0.001 for interaction of humidity and development) (Figure 2A). The increase in wet body mass during development was not different between Control and High RH groups, but mean wet body mass of Low RH group was significantly lower than that of Control and High RH groups during the last 3 days of incubation (d17-19). Mean wet body mass at hatch was 43.20 ± 0.71 g (Control), 40.65 ± 1.07 g (Low RH) and 46.09 ± 1.18 g (High RH), which differed significantly among the three groups (*p* = 0.006). Pairwise multiple comparisons indicated a significant difference between Low and High RH groups (*p* < 0.005). At hatchling euthanasia on d22, mean wet body mass had decreased to 37.02 ± 1.01, 35.73 ± 1.08 and 38.55 ± 1.42 g for of Control, Low and High RH groups, respectively, with no significant difference between them (*p* = 0.271).

#### 3.3.2 Dry Body Mass

Changes in dry body mass were significant across development (*p* < 0.001), but were not significantly different between the three RH groups (*p* = 0.695 for effect of humidity), with no significant interaction of development and humidity (*p* = 0.877) (Figure 2A). The mean dry body mass at hatching was 9.44 ± 0.24, 9.41 ± 0.33 and 9.62 ± 0.36 g for Control, Low and High RH groups, respectively, and the difference between RH groups was not significant (*p* = 0.876).

### 3.4 Body Water Content

Daily changes in water content of embryos were significant across development (*p* < 0.001) and between the three RH groups (*p* < 0.001), with significant interaction of development and RH (*p* < 0.001) (Figure 2B). The daily changes of water content were not different between Control and High RH groups, but mean water content of Low RH group was significantly lower than that of High RH group during d16-19 and that of Control group on d19 of incubation.

### 3.5 Hematology

**FIGURE 3:**
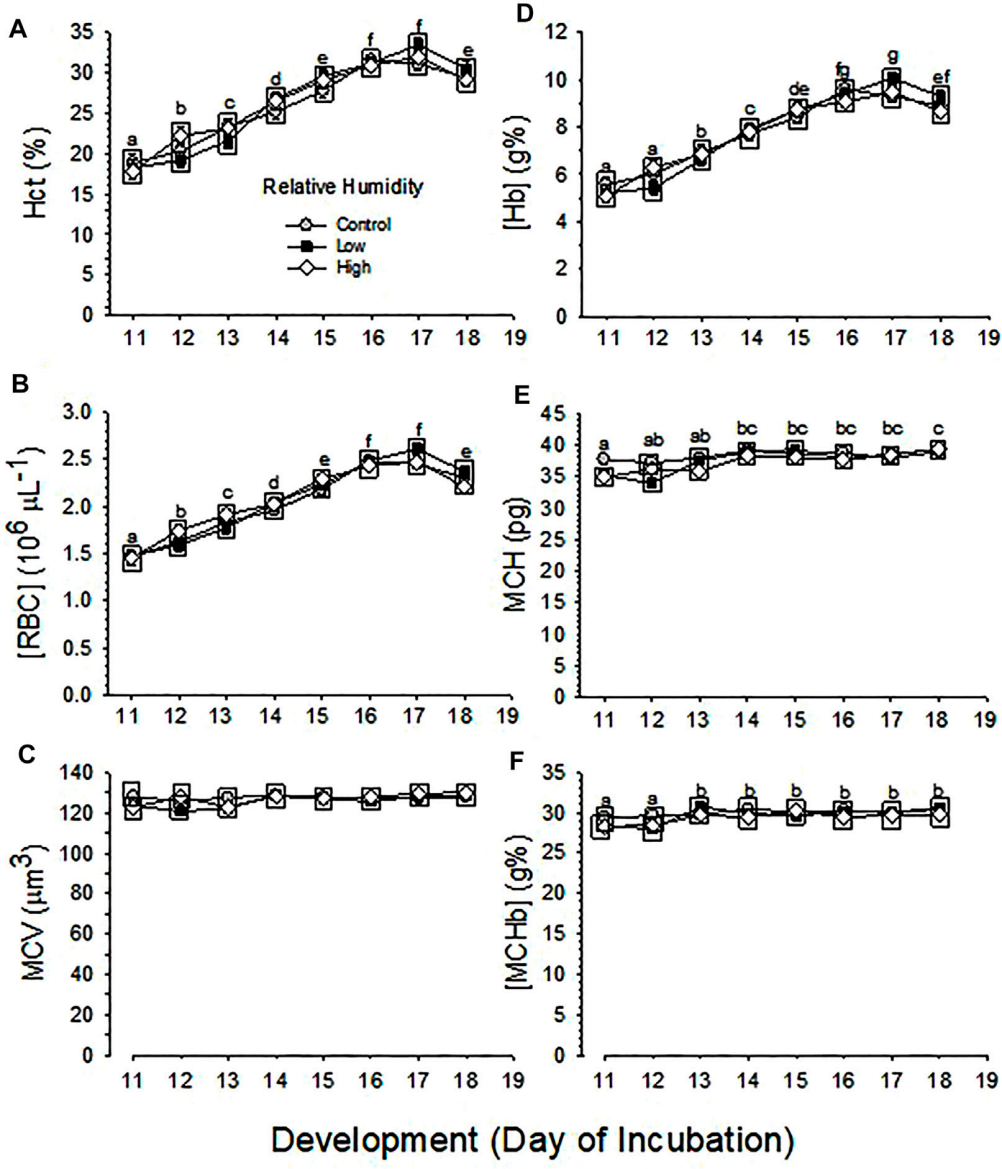
Developmental changes in hematological variables of embryos incubated at Control, Low and High relative humidity. Means ± standard error are presented. Error bars are smaller than the symbol size. Different lowercase letters indicate significant differences across development. Boxes enclosing statistically identical means are omitted for clarity. **(A)** Hct **(%)** is not significantly (*p* = 0.616) affected by RH (vertical comparison), but is significantly affected by day of development (*p* < 0.001), with no significant interaction between the two variables (*p* = 0.365). Different lowercase letters indicate significant differences across development. **(B)** [RBC] is not significantly (*p* = 0.539) affected by RH (vertical comparison), but is significantly affected by development (*p* < 0.001), with no significant interaction between the two variables (*p* = 0.395). Different lowercase letters indicate significant difference. **(C)** MCV is not significantly affected by RH (*p* = 0.106) or development (*p* = 0.054) and their interaction is not significant (*p* = 0.508). **(D)** [Hb] is not significantly affected by RH (*p* = 0.799), but is affected by development (*p* < 0.001), with no significant interaction (*p* = 0.226) between the two variables. **(E)** MCH is significantly affected by both RH (*p* = 0.011) and development (*p* < 0.001), and the interaction between these two variables is significant (*p* = 0.036). **(F)** [MCHb] is significantly affected by both humidity (*p* = 0.027) and development (*p* < 0.001), with no significant interaction (*p* = 0.661) between the two variables. n values are as in Figure 1.

Developmental changes in Hct and [RBC] were not different between the three RH groups (*p* = 0.616 and *p* = 0.539 for effect of humidity on Hct and [RBC], respectively), but were significant across development (*p* < 0.001) for both Hct and [RBC]), with no significant interaction between humidity and development (*p* = 0.365 and *p* = 0.395 for Hct and [RBC]) (Figures 3A,B).

Changes across development in MCV were not different between the three RH groups (*p* = 0.106 for effect of humidity), nor were significant across development (*p* = 0.054) with no significant interaction (*p* = 0.508) (Figure 3C).

Changes across development in [Hb] were significant (*p* < 0.001), but were not different between the three RH groups (*p* = 0.799), with no significant interaction (*p* = 0.226) (Figure 3D).

Changes across development in MCH and [MCHb] were significant (*p* < 0.001) and these variables different significantly between the three RH groups (*p* = 0.011 and *p* = 0.027 for MCH and [MCHb], respectively), with significant interaction for MCH (*p* = 0.036) or with no significant interaction for [MCHb] (*p* = 0.661) (Figures 3E,F).

### 3.6 Blood Chemistry

#### 3.6.1 Osmolality

**FIGURE 4:**
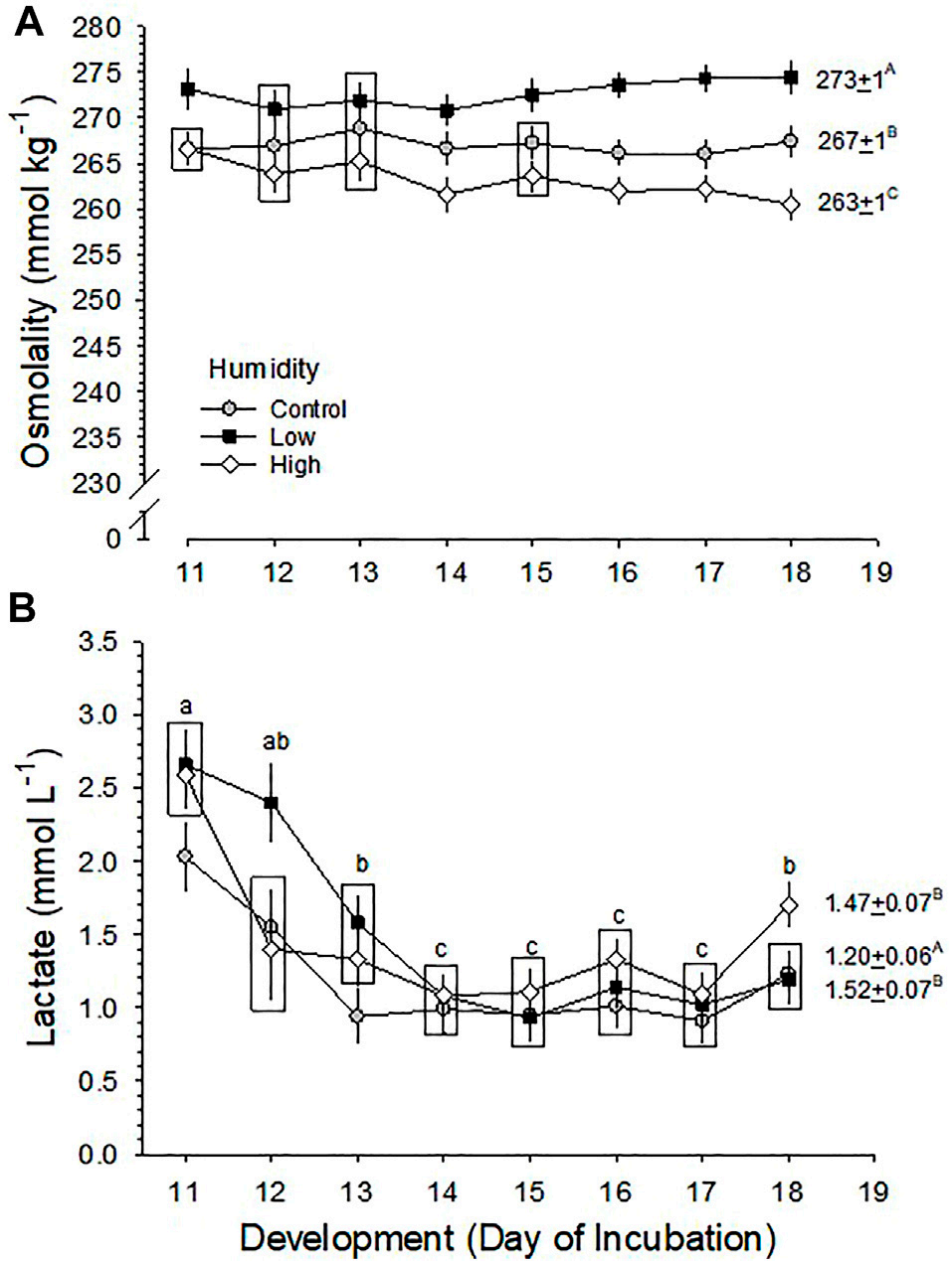
Developmental changes in **(A)** blood osmolality (Osm) and **(B)** [Lactate] in embryos incubated at Control, Low and High relative humidity. Means ± standard error are presented. Means on any given day of incubation that are not significantly different from each other are enclosed by a box. **(A)** Blood Osmolality: The effect of RH on Osm (vertical comparison) is significant (*p* < 0.001), but effect of development (*p* = 0.787) and their interaction (*p* = 0.671) are not significant. Mean Osm across development is shown to the right of each RH plot, and these values are significantly different from each other (*p* < 0.001 for any two comparisons), as indicated by uppercase letters. **(B)** [La^−^]: [La^−^] was significantly affected by both humidity (*p* = 0.001) and development (*p* < 0.001), with no significant interaction (*p* = 0.060). Mean [La^−^] during the last half of incubation (D11-D19) is shown to the right of each RH plot, with uppercase letters indicating differences between means. Mean [La^−^] values of Low and High RH groups are significantly higher than the Control value (*p* = 0.002 for Low RH *vs.* Control RH, *p* = 0.010 for High RH *vs*. Control RH, *p* = 0.610 for Low RH *vs*. High RH). Lowercase letters indicate significant differences across development. *n* values are as in Figure 1.

Blood osmolality (Osm) did not change significantly across development in the three RH groups (*p* = 0.787), but mean values of Osm were significantly different between the three groups (*p* < 0.001 for effect of humidity), with no significant interaction of development and humidity (*p* = 0.671) (Figure 4A). The mean Osm during the period d11-18 was 273 ± 1, 267 ± 1 and 263 ± 1 mmol kg^−1^ for Low, Control and High RH, respectively.

#### 3.6.2 Lactate Concentration

Blood lactate ion concentration ([La^−^]) changed significantly across development in all three RH groups (*p* < 0.001). Mean values of [La^−^] were significantly different between three RH groups (*p* = 0.001) with no significant interaction of development and humidity (*p* = 0.060), but there was no clear pattern of change associated with incubation RH (Figure 4B). The mean [La^−^] of Low and High RH groups were 1.52 ± 0.07 mmol L^−1^ and 1.47 ± 0.07 mmol L-1, respectively, which were not different from each other, but were both significantly higher than that of the Control group (1.20 ± 0.06 mmol L^−1^).

#### 3.6.3 Blood gas Pressures and Acid-Base Balance

**FIGURE 5:**
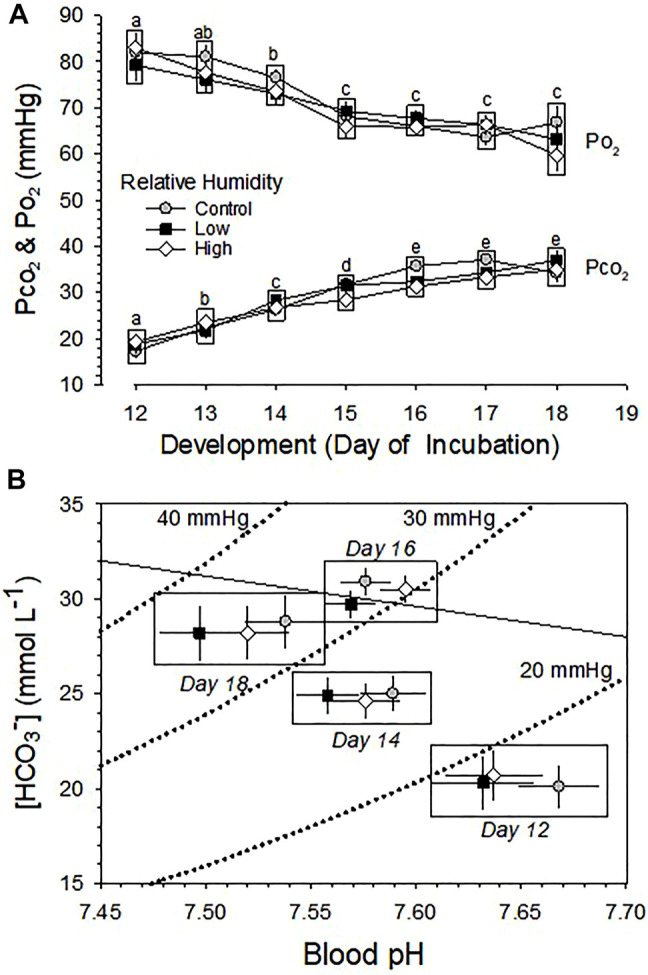
Developmental changes in blood gas pressures and acid-base balance in embryos incubated at Control, Low and High relative humidity. Means ± standard error are presented. In some cases error bars are smaller than the symbol size. Means on any given day of incubation that are not significantly different from each other are enclosed by a box. **(A)** Arterialized blood Po_2_ and Pco_2._ Arterialized blood Po_2_ and Pco_2_ are not significantly affected by RH (*p* ≥ 0.359). Means on any given day of incubation that are not significantly different from each other (vertical comparison) are enclosed by a box. However, there is a significant effect of development on both Po_2_ (*p* < 0.001) and Pco_2_ (*p* < 0.001), with no significant interactions (*p* = 0.691 for Po_2_ and *p* = 0.134 for Pco_2_). Different lowercase letters indicate significant difference between development. **(B)** Acid-base balance. For clarity, acid-base variables for incubation days 13, 15 and 17 are omitted from this Davenport diagram. RH did not significantly affect blood pH (*p* = 0.133) or blood [HCO_3_^−^] (*p* ≥ 0.241), but there was a significant effect of development on pH (*p* < 0.001) and [HCO_3_^−^] (*p* < 0.001), with no significant interaction between these variables (*p* = 0.491 for pH and *p* = 0.781for [HCO_3_^−^]). n values are as in Figure 1.

Changes across development in arterialized blood Po_2_ and Pco_2_ were both significant (*p* < 0.001), but were no significant differences between the three RH groups (*p* > 0.35) and no significant interaction between development and humidity (*p* = 0.691 and *p* = 0.134 for Po_2_ and Pco_2_, respectively) (Figure 5A).

Changes across development in the pH and [HCO_3_^−^] of arterialized blood were both significant (*p* < 0.001), but were not significant between the three RH groups (*p* > 0.136), with no significant interaction between development and humidity (*p* > 0.49) **(** Figure 5B).

### 3.7 Hematological and Acid-Base Disturbances Associated With Hypoxemia and Hypercapnia

#### 3.7.1 Hematological Variables

**FIGURE 6:**
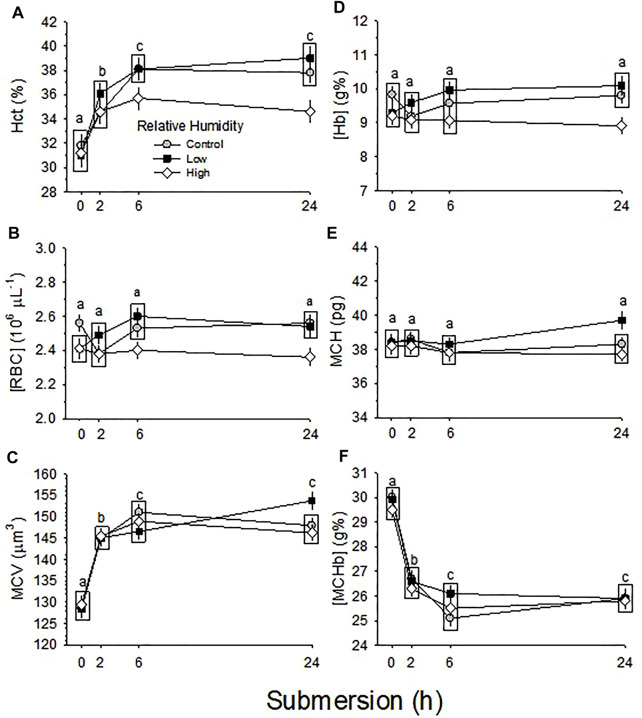
Hematological changes in response to 24 h partial water submersion at d17 of incubation. Means ± standard error are presented. In some cases error bars are smaller than the symbol size. Means on any given day of incubation that are not significantly different from each other are enclosed by a box. Different lowercase letters indicate significant difference between submersion times. **(A)** Hct **(%).** Hct was significantly affected by both RH (*p* = 0.005) and partial water submersion (*p* < 0.001), with no significant interaction (*p* = 0.232). **(B)** [RBC]. [RBC] was significantly affected by RH (*p* = 0.001) but not partial water submersion (*p* = 0.185), with insignificant interaction (*p* = 0.179) occurring between the two variables. **(C)** MCV. MCV was not significantly affected by RH (*p* = 0.780), but was significantly affected by submersion (*p* < 0.001), with no significant interaction between the two variables (*p* = 0.096). **(D)** [Hb]. [Hb] was significantly affected by humidity (*p* < 0.001) but not partial water submersion (*p* = 0.438), with no significant interaction (*p* = 0.226) between the two variables. **(E)** MCH. MCH was not significantly affected by either RH (*p* = 0.072) or submersion (*p* = 0.419), with no significant interaction (*p* = 0.463) between the two variables. **(F)** [MCHb]. [MCHb] was significantly affected by submersion (*p* < 0.001) but not by RH (*p* = 0.333), with no significant interaction (*p* = 0.550) between the two variables. *n* values for each condition and each hour of submergence are indicated in Figure 8.

In response to half submersion of eggs in water with egg air cell down, Hct significantly increased (*p* < 0.001) in all three RH groups (*p* = 0.005) (Figure 6A). Mean Hct during submersion was 35.6 ± 0.5, 36.1 ± 0.5 and 34.0 ± 0.5% for Control, Low and High RH groups, respectively. The increase in High RH group was significantly less compared with the increases in Control and Low RH groups, which did not differ from each other. However, the extent of increase in Hct with submersion length was not different between the three RH groups (*p* = 0.232) (Figure 6A).

[RBC] in the three RH groups was not significantly changed by any length of submersion (*p* = 0.185) (Figure 6B). However, mean [RBC] during submersion (2.51 ± 0.03, 2.51 ± 0.03 and 2.39 ± 0.03 × 10^6^ µL^−1^ for Control, Low and High RH groups, respectively), was significantly different (*p* = 0.001). [RBC] of High RH group was significantly lower compared with [RBC] of Control and Low RH groups, which were not different. The significant difference in [RBC] between the three RH groups was not changed by submersion (*p* = 0.179).

MCV significantly increased with submersion time (*p* < 0.001) in all three RH groups, with no significant difference of magnitude of increase between groups (*p* = 0.780) (Figure 6C). The interaction between effects of RH and submersion was not significant (*p* = 0.096).

[Hb] was not significantly changed by any length of submersion (*p* = 0.438) in the three RH groups, but mean [Hb] during submersion; 9.6 ± 0.1, 9.7 ± 0.1 and 9.1 ± 0.1 g% for Control, Low and High RH groups, respectively, was significantly different (*p* < 0.001) (Figure 6D). [Hb] of High RH group was significantly low compared with [Hb] of Control and Low RH groups that were not different. There was no significant interaction between the three RH groups for submersion (*p* = 0.226 for interaction) (Figure 6D).

MCH was not changed by any length of submersion (*p* = 0.419) and mean MCH was not different between the three RH groups (*p* = 0.072) with no significant interaction between effects of RH and submersion (*p* = 0.463) (Figure 6E).

[MCHb] was significantly decreased by at all submersion times (*p* < 0.001) in the three RH groups, but there was no significant difference of magnitude between RH groups (*p* = 0.333) (Figure 6F). The interaction between RH and submersion was not significant (*p* = 0.550).

**FIGURE 7:**
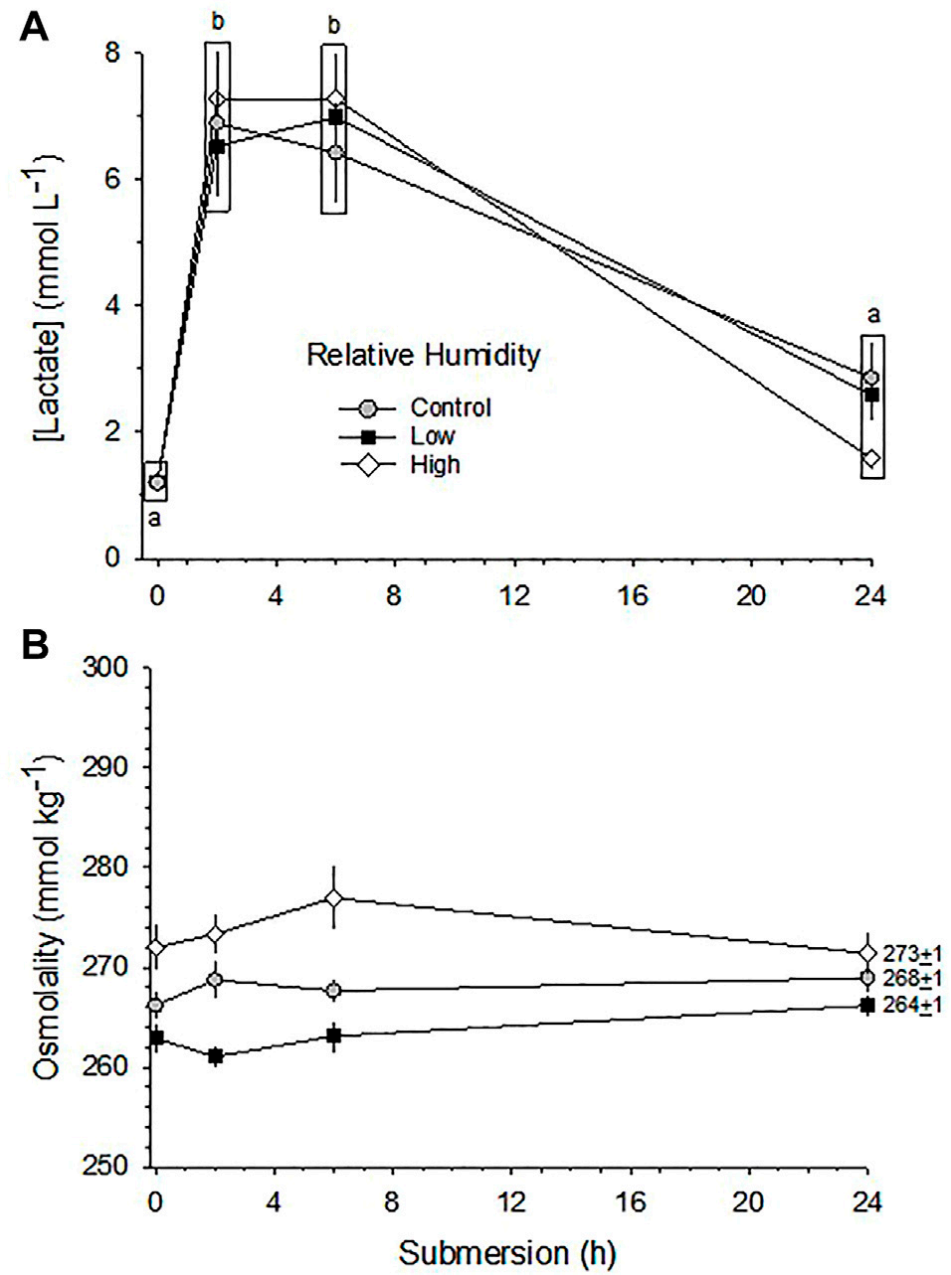
Changes in [La^−^] **(A)** and [Osm] **(B)** in response to 24 h partial water submersion at d17 of incubation. Means ± standard error are presented. Means on any given day of incubation that are not significantly different from each other are enclosed by a box. Different lowercase letters indicate significant difference between submersion times. n values for each condition and each hour of submergence are indicated in Figure 8.

[La^−^] was significantly affected by submersion in three RH groups (*p* < 0.001). [La^−^] increased sharply after just 2 h of submersion in all three humidity groups and stayed elevated through hour 6. By hour 24, however, [La^−^] had declined in the Control and Low RH groups, but were still significantly elevated in the High RH group (Figure 7A).

While the differences in [Osm] due to relative humidity differences was maintained throughout the submersion period, in no group did submersion *per se* significantly affect [Osm] (*p* > 0.54) (Figure 7B).

#### 3.7.2 Acid-Base Balance

**FIGURE 8:**
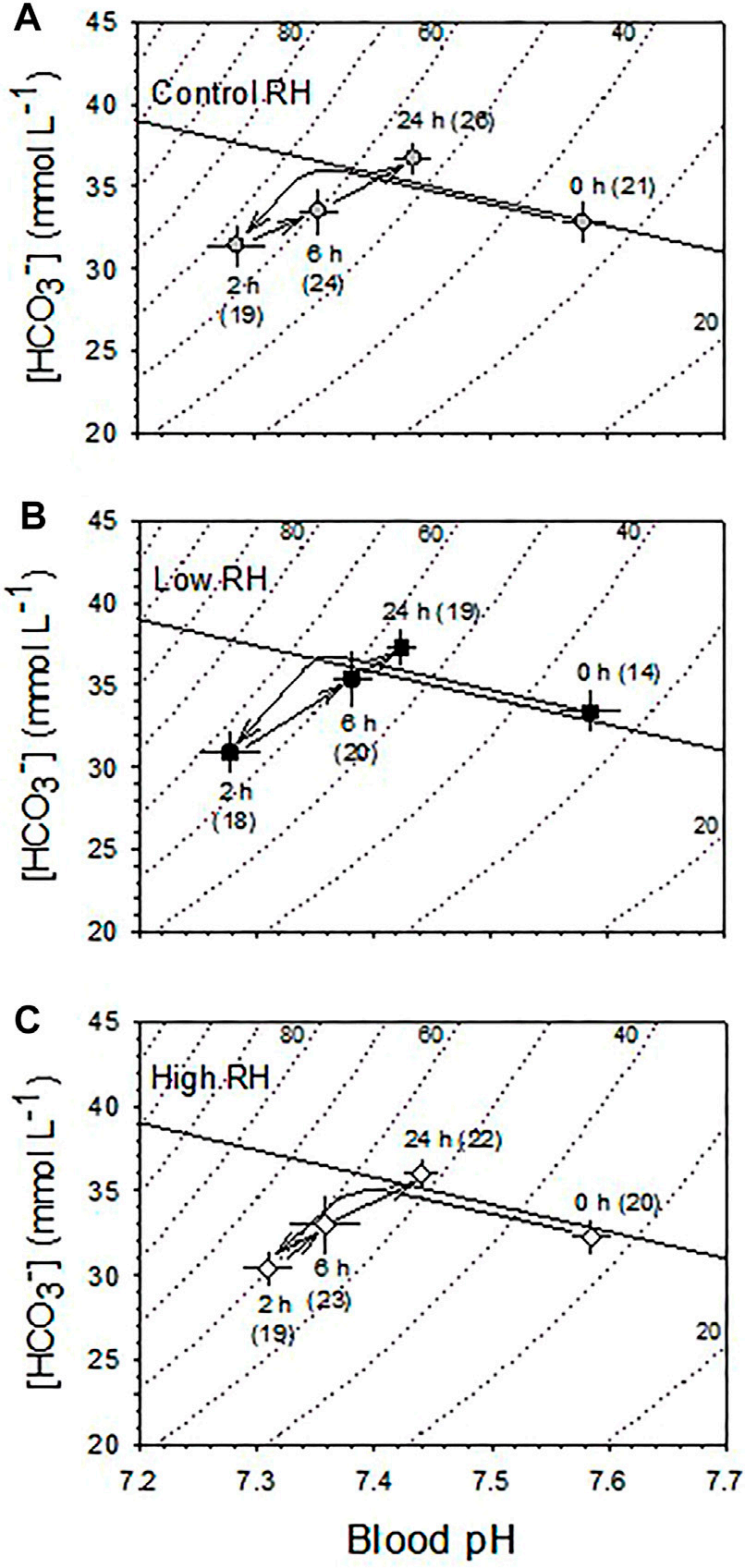
Blood acid-base balance changes during the course of 24 h partial water submersion at d17 of incubation. **(A)** Control, **(B)** Low and **(C)** High humidity. Means ± standard error are presented in this Davenport Diagram. N values are indicated in parentheses. Measurements were made at 0, 2, 6, and 24 h following partial water submersion. Arrows indicate direction of change in mean values during the course of submersion. Both pH (*p* < 0.001) and [HCO_3_^−^] (*p* < 0.001) were significantly affected by partial water submergence in all three RH groups. However, there was no significant effect of incubation RH on either blood pH (*p* = 0.762) or [HCO_3_^−^] (*p* = 0.359), and there was no significant interaction between RH and submersion hour (*p* > 0.87). n values indicated in parentheses.

Arterialized blood pH and [HCO_3_^−^] of all three RH groups were significantly different across time submerged in water (*p* < 0.001) (Figure 8). However, there were no significant differences (*p* > 0.36) between the responses of the RH groups at any point during submersion with no significant interaction between effects of RH and submersion time (*p* > 0.86). All three RH groups initially showed an immediate mixed metabolic and respiratory acidosis at 2h, with blood PCO_2_ before submersion (35 ± 1) rising significantly by ∼30 mmHg–68 ± 3 mmHg. Concurrently, the pH before submersion, (7.58 ± 0.2), fell ∼0.3 units to 7.28 ± 0.1 at 2 h. However, from 2 to 24 h there was a partial compensation of the metabolic and respiratory acidosis, with PCO_2_ declining significantly from its peak by ∼10 mmHg–56 ± 2 mmHg over this period and pH rising by ∼0.15 mmHg–7.42 ± 0.1.

## 4 Discussion

### 4.1 Hatchability

The hatching success of eggs incubated in commercial incubators is determined, in part, by incubator relative humidity. The relationship between embryo mortality and RH has been empirically determined and used to generate a bimodal curve (Ar and Rahn, 1980). In the literature on chicken egg incubation, effects of incubation RH on hatchability vary somewhat. For example, in several studies the RH for optimum hatchability ranged from ∼40 to 65%, maximum hatchability occurred at 50% RH, and hatchability was decreased at ∼35–20% RH (Robertson I. S., 1961; Snyder and Birchard, 1982; Tullet and Burton, 1982; van der Pol et al., 2013). In contrast, there were no differences in hatchability among eggs incubated at ∼40–70% RH (Buhr, 1995) and, similarly, in our study mean hatchability was unaffected by Low or High RH. There are clear physiological differences between different strains of chickens (Flores-Santin and Burggren, 2021) and these differences may extend back to the embryo, perhaps accounting for hatching variability in different relative humidities. Further experiments are required to determine the mechanisms underlying humidity-affected hatchling success.

### 4.2 Body Mass

Although RH did not affect hatchability in the current experiment, hatchling body mass was altered, with body mass increasing in proportion to incubation RH. Again, results of RH differences in the literature are variable. There was no effect of RH on wet body mass of hatchlings hatched from eggs incubated at 40 and 70% RH and wet body mass at hatch was not different between Control and 85–90% RH groups (Robertson I. S., 1961; Davis et al., 1988). Wet body mass at hatch decreased in eggs incubated at 20 and 33% RH and increased with increasing RH (Tullet and Burton, 1982; Peebles et al., 1987; Hamdy et al., 1991; Bruzual et al., 2000). Bruzual et al. (2000) reported that wet body mass at hatch increased significantly with increasing RH treatment, but BM at pull (removal from machine) was not different. That result resembles as our observations. Dry body mass was unaffected by RH in the current study (Figure 2A) as well as that of Tullet and Burton (1982). Collectively, these data suggest that variation in wet body mass under different conditions results from hydration/dehydration of the embryos, rather than actual changes in growth.

Since dry body mass of embryos was not different between the three groups with larger body water content in Control and High RH groups compared with Low RH group, embryos in Control and High RH groups are assumed to be becoming hydrated late in incubation toward hatching.

### 4.3 Blood Variables

Blood Osm reflected tissue water content as influenced by incubation RH, as expected, with blood Osm being ∼4% higher in Low RH compared to High RH groups (Figure 4A). Similarly, lactate concentration was different between RH groups at different points in development (Figure 4B). Increased blood [La^−^] has traditionally been viewed to be the result of stress, particularly if aerobic metabolism is affected, although increasingly lactate is viewed as an intermediary in aerobic metabolism, as well (Brooks, 2018, 2020). In the present study, the highest lactate levels were found in the High RH group later in development. Since blood Po_2_ was unaffected by RH (Figure 5), it is unlikely that changes in glycolysis under different incubation RHs was contributing to any changes in [La^−^]. Noteworthy is that no consistent patterns emerged during incubation and even the largest [La^−^] differences were only in the order of 50–70%, compared to as much as 16-fold increases that can occur in chicken embryos when experiencing hypoxia (Tazawa et al., 2012).

Developmental changes in hematological respiratory variables in the developing chicken embryo have been reported in many studies (Yosphe-Purer et al., 1953; Barnes and Jensen, 1959; Temple and Metcalfe, 1970; de W Erasmus et al., 1970; Tazawa, 1971; Tazawa et al., 1971a, 1971b; Tazawa, 1984; Dzialowski et al., 2002; Black and Burggren, 2004; Khorrami et al., 2008; Bolin et al., 2017). Some studies and reviews summarizing several reports indicated that hematological variables plateaued towards the end of incubation (Johnston, 1955; Macpherson and Deamer, 1964; Jalavisto et al., 1965; Romanoff, 1967; Tazawa, 1980; Davis et al., 1988; Tazawa et al., 2011). Although the current study did not collect arterialized blood after d19, Hct, [RBC] and [Hb] reached maximal values on d16-17 with subsequent decreases on d18 of development (Figure 3), rather than plateauing. This decrease is consistent with other studies showing a temporary decrease in red blood cells and related hematological variables prior to hatching (Clark, 1951; Bartels et al., 1966; Ackerman, 1970; Freeman and Misson, 1970; Lemez, 1972). The reason for decreases of Hct, [RBC] and [Hb] remain to be studied.

There was no influence of incubation RH on hematological variables (Figure 3 **),** on blood PO_2_, PCO_2_ and acid-base balance variables (Figure 5 **).** Gas diffusion conductance of the eggshell and shell membranes remains unchanged during the last half of incubation when the chorioallantoic membrane has fully developed, so arterialized blood Po_2_ decreases and Pco_2_ increases due to increasing metabolism of developing embryos (Figure 5A). Simultaneously, as dissolved CO_2_ increases in blood with development of embryos, acid-base status of older embryos develops a respiratory acidosis relative to younger embryos (Figure 5B). Yet, these significant developmental changes in blood gases and acid-base balance were completely unaffected by incubation RH, even as water content and body mass were greatly affected.

### 4.4 Incubation RH and Regulation of Hematological and Acid-Base Balance

Partial water submersion of embryonated eggs is a useful tool for decreasing gas exchange through the eggshell, resulting in intrinsic O_2_ deficiency (hypoxemia) and CO_2_ accumulation (hypercapnia) in the embryo (Andrewartha et al., 2014; Kohl et al., 2015; Branum et al., 2016). In the current study, hypoxemia and hypercapnia induced swelling of the red blood cells as a result of hypoxemia and hypercapnia—i.e. Hct and MCV both increased. The maximum change was typically reached at 6 h of submergence, with no further change through to 24 h of submergence (Figure 6). Interestingly, no hematological variables at the 6 h point of submersion differed between the Control and Low RH groups. Notably, however, the High RH group at 6 h and especially at 24 h had a significantly lower Hct, lower [Hb], lower [RBC] compared with the Control and Low RH groups, which still showed a greater disruption caused by hypoxemia and hypercapnia. That is, the High RH group at 6 and especially 24 h showed the smallest deviation from pre-submergence values of all hematological variables (Figure 6). Thus, we posit that the additional tissue water content associated with High RH incubation provides some degree of protection against hematological disturbance induced by submergence-related intrinsic hypoxemia and hypercapnia. Identification of the mechanism affording this red blood cell protection awaits further experimentation.

The initial development of large mixed metabolic and respiratory acidosis of the blood upon partial water submergence was unaffected by incubation RH, and was reflected by the very large and immediate increase in blood [La^−^] (Figure 7). Similarly, the partial recovery through primarily metabolic mechanisms after 2 h of submergence developed to the same extent in all three RH groups (Figure 8). These data suggest that the regulatory capability to at least partially restore blood acid-base balance exists independently of the severe disturbances to tissue water content caused by incubation in High or Low RH. To what extent other physiological systems—e.g., renal, cardiovascular—retain the ability to carry out their physiological functions awaits additional experimentation.

### 4.5 Implications of Non-Ideal Incubation Humidity

This study has revealed that chronic relative humidities above or below optimum can to some extent influence both morphological and physiological factors at the time of hatching. Several questions arise: Do hatchlings showing alterations at birth as a result of incubation in non-ideal humidity subsequently ‘grow out of’ these modifications into adulthood, or are they retained as life-long effects? Do hatchlings with normal morphological and physiological variables despite Low and High RH-incubation actually develop abnormalities during subsequent development to adulthood? While entirely speculative could there be effects of RH incubation that, through epigenetic inheritance, carry over into the offspring of chickens incubated in non-ideal conditions? Certainly, in the King quail (*Coturnix chinensis*) there can be transgenerational effects of pollutant exposure (Bautista et al., 2021). Could this also be the case for RH-induced changes on the embryo? Finally, could different breeds of chicken have different response to incubation RH, and might they thus be better adapted to low or high humidity climates? Answering these and other questions warrants additional study in chickens and other birds.

## Data Availability

The raw data supporting the conclusions of this article will be made available by the authors, without undue reservation.
